# Prognostic role of multiparameter MRI and radiomics in progression of advanced unresectable hepatocellular carcinoma following combined transcatheter arterial chemoembolization and lenvatinib therapy

**DOI:** 10.1186/s12876-022-02129-9

**Published:** 2022-03-08

**Authors:** Junpeng Luo, Zhimei Huang, Murong Wang, Tian Li, Jinhua Huang

**Affiliations:** 1 Department of Minimally Invasive Interventional Radiology, Sun Yat-Sen University Cancer Center, State Key Laboratory of Oncology in South China, Collaborative Innovation Cancer for Cancer Medicine, 651 Dongfeng Road East, Guangzhou, 510060 Guangdong Province People’s Republic of China; 2 School of Basic Medicine, Fourth Military Medical University, No. 169 Changle West Rd, Xi’an, 710032 People’s Republic of China

**Keywords:** Hepatocellular carcinoma, Lenvatinib, Chemoembolization, Magnetic resonance imaging, Radiomics, Progression free survival

## Abstract

**Background:**

Current study aims to determine the prognostic value of Multiparameter MRI after combined Lenvatinib and TACE therapy in patients with advanced unresectable hepatocellular carcinoma (HCC).

**Methods:**

A total of 61 HCC patients with pre-treatment Multiparameter MRI in Sun Yat-sen University Cancer Center from January 2019 to March 2021 were recruited in the current study. All patients received combined Lenvatinib and TACE treatment. Potential clinical and imaging risk factors for disease progression were analyzed using Cox regression model. Each patient extracts signs from the following 7 sequences: T1WI, T1WI arterial phase, T1WI portal phase, T1WI delay phase, T2WI, DWI (b = 800), ADC.1782 quantitative 3D radiomic features were extracted for each sequence, A random forest algorithm is used to select the first 20 features by feature importance. 7 logit regression-based prediction model was built for seven sequences based on the selected features and fivefold cross validation was used to evaluate the performance of each model.

**Results:**

CR, PR, SD were reported in 14 (23.0%), 35 (57.4%) and 7 (11.5%) patients, respectively. In multivariate analysis, tumor number (hazard ratio, HR = 4.64, 95% CI 1.03–20.88), and arterial phase intensity enhancement (HR = 0.24, 95% CI 0.09–0.64; P = 0.004) emerged as independent risk factors for disease progression. In addition to clinical factors, the radiomics signature enhanced the accuracy of the clinical model in predicting disease progression, with an AUC of 0.71, a sensitivity of 0.99%, and a specificity of 0.95.

**Conclusion:**

Radiomic signatures derived from pretreatment MRIs could predict response to combined Lenvatinib and TACE therapy. Furthermore, it can increase the accuracy of a combined model for predicting disease progression. In order to improve clinical outcomes, clinicians may use this to select an optimal treatment strategy and develop a personalized monitoring protocol.

## Introduction

Liver cancer is the sixth most common cancer diagnosed worldwide and the third leading cause of cancer death [1, 2]. Primary liver cancers include hepatocellular carcinoma (HCC) (representing 75–85%) and intrahepatic cholangiocarcinoma (representing 10–15%), among others. Although radical resection is an intervention against HCC, it is unsuitable in 50–70% of HCC patients due to liver dysfunction, late stage of the tumor or poor performance.

Transcatheter arterial embolization (TAE), also referred to as transcatheter arterial chemoembolization (TACE) has become the standard treatment for unresectable or hypervascular tumors such as hepatocellular carcinoma (HCC) [3]. In addition, the previous literature has proved that in patients with liver cancer, bland embolization has no advantage over transarterial chemoembolization [4].Therefore, the conventional TACE has a wider application range, and the research in this article is also based on this. Previous studies have established that Lenvatinib, a multi-kinase inhibitor with anti-angiogenic effects, is as efficacious as Sorafenib in first-line treatment of advanced-stage HCC [5]. Majority of advanced-stage HCC patients still have poor prognosis despite recent advances in treatment, with median survival time remaining at approximately one year [6]. Recent studies have reported excellent therapeutic results with combined TACE and Lenvatinib treatments [7–9]. These outcomes were superior to those of Lenvatinib monotherapy and TACE alone. Research on reliable non-invasive biomarkers to predict effects of combination therapy should be undertaken. This will result in identification of suitable candidates for treatment of advanced stage HCC.

Because of multiparameter magnetic resonance imaging (MP-MRI)'s high soft-tissue resolution and non-ionizing radiation, non-invasive evaluation of HCC has become practical and affordable [10, 11]. Further, image-based radiomics signatures can be used to assess prognosis of multiple tumors using MRI, making them a noninvasive, affordable method for evaluating HCC. Recently, MRI has shown to be effective in assessing the efficacy of both TACE and radiofrequency ablation in HCC patients. However, A combination of lenvatinib and TACE for the treatment of HCC based on MRI radiomics has not been evaluated.

Using multiparameter MRI imaging, the present study examined the role of pretreatment MRI in predicting efficacy of combined Lenvatinib and TACE therapy in advanced unresectable HCC.

## Methods and materials

### Study population

This retrospective study was approved by Ethical Review Board of Sun Yat-sen University Cancer Center and informed consent was waived (B2019-160). A total of 61 patients who underwent combined TACE and Lenvatinib therapy at the medical center of the University between January, 2019 and March, 2021 were included in the current study. Inclusion criteria included: (1) Consent that has been signed and informed; (2) BCLC stage A or B or C; (3) patients with initial treatment with combined TACE and Lenvatinib therapy; and (4) Within one or two weeks prior to treatment, patients had a dynamic contrast enhanced MRI.

On the other hand, exclusion criteria included: (1) Expected survival period of 3 months; (2) Refusal to participate in the study; and (3) Patients with follow-up time of < 2 months.

### TACE procedure

Patients received transcatheter arterial chemoembolization (TACE) from experienced interventional radiologists (IRs). In a 5-F angiographic catheter, angiograms of celiac trunk and superior mesenteric artery were performed under local anesthesia with a diagnostic angiographic catheter (RH or Yoshiro catheter; Terumo, Tokyo, Japan). An artery feeding the tumor was identified with a 2.7-F microcatheter (Terumo, Tokyo, Japan) during super selective catheterization. Emulgator mixed with lipiodol (specification: 10 mg/bottle, Jiangsu Hengrui Pharmaceutical Co., Ltd.China) and chemotherapy drugs were then administered. Chemotherapy drugs used in the current study included Lobaplatin (10 mg/bottle, Hainan Changan International Pharmaceutical Co., Ltd.China) and Pirarubicin hydrochloride (10 mg/bottle, Hanhui Pharmaceutical Co., Ltd.China). Chemoembolization was undertaken using iodized oil 5–20 mL mixed with Lobaplatin and Pirarubicin hydrochloride. We used the chemotherapeutic-in-oil (CiO) technique [12].Observation of tumor stain after administration of scheduled dose would necessitate use of gelatin sponge or PVA particles for embolism.

### Combination therapy with Lenvatinib

TACE and Lenvatinib were recommended as a treatment strategy, and patients were fully informed regarding the benefits, risks, and costs of the drugs. Three days after the first TACE treatment, Lenvatinib was administered to patients after their consent. Patients who declined Lenvatinib treatment were only treated with TACE. Lenvatinib was administered once daily at a dosage of 12 mg (≥ 60 kg) or 8 mg (< 60 kg). Treatment with Lenvatinib was stopped three days prior to the start of TACE sessions and restarted after each TACE session provided that the patients didn't show TACE-related adverse effects.

Lenvatinib-related toxicities were reduced by interrupting therapy and then decreasing doses (8 mg and 4 mg/day, or 4 mg every other day).

### Anti‑viral therapy

Antiviral therapy (Tenofovir or Entecavir) was administered before treatment to patients with hepatitis B virus (HBV) infection, and continued during the long-term therapy. Follow up included monitoring the viral load. Sofosbuvir therapy was administered from the time of baseline to patients who had hepatitis C virus infection.

### Assessment of safety

Adverse events (AEs) related to treatment were evaluated mainly by their frequency and severity based on the Common Terminology Criteria for Adverse Events(CTCAE, version 5.0) [13]. Adverse events during follow-ups of all patients were recorded at intervals of 6–8 weeks. Any transient side effects occurring just after TACE, including fever, abdominal pain, or elevated liver enzyme levels (such as aspartate transaminase (AST)/alanine aminotransferase (ALT)) were not recorded.

### Follow-up surveillance

Based on the Modified Response Evaluation Criteria in Solid Tumors (mRECIST) [14], progressive disease is defined as an increase of at least 20% in viable (enhancing) lesions, the smallest diameters of viable (enhancing) lesions measured during the treatment period is taken as the reference. Every month after the first year and every 3 months thereafter, patients were followed up with serum α-fetoprotein level and liver function tests as well as chest and abdomen contrast-enhanced CT scans and contrast-enhanced MRI exams. Data were censored on June 13, 2021. In computing progression-free survival, the time since the beginning of treatment was divided by the time since the first progression, metastasis, or last follow-up.

### MRI protocol

All MR images were taken using three devices: United Imaging Healthcare, Shanghai, China; and Siemens, Erlangen, Germany; GE, Boston, United States. The type of contrast media is Gd-EOB-DTPA [15]. Table 3 provides detailed information about scanning parameters about T1 and T2 series. We exported the MRI sequences as DICOM files.

### Image features

The following MRI image characteristics were evaluated; (1) location (Near or not the hepatic hilar); (2) Margins of tumors (smooth or irregular); (3) types of gross growth (simple nodulation with distinct margins, extra-nodular growth of the lobular type, multiple nodular growth at the intersection, and invasion border with irregular growth); (4) Apparent Diffusion Coefficient (ADC) intensity display (iso-hyperintensity, hypointensity); (5) Homogeneity of signal on T2WI (homogenous, heterogeneous); (6) The arterial phase shows greater or equal intensities than the portal phase (marked: higher than the portal phase, mild: lower than the portal phase); (7) Extension of arterial phase range; (8) type of enhancement (progressive: intensity or range increased over time, persistent: enhancement remained constant throughout the three phases, degressive: hyperintensity decreased over time without hypointensity on delay or portal phases); (9) Peritumoral arterial enhancement: An increase in hyperintensity with fuzzy margins and isointensity with normal liver tissue upon further dynamic changes (yes or no); (10) Enlarging the tumor capsule (completely or partially); (11) hepatic capsule bulge (yes, or no); (12) scarring centrally (yes, or no); (13) hemorrhage (yes, or not); 14) necrosis (yes or not); (15) fat (yes, or no); (16) massive vein tumor thrombosis (yes, or no); and (17) metastasis in distant areas (yes, or no).

Two independent radiologists (each one has 7 years of work experience) evaluated images separately, followed by assessment of interobserver agreement [16]. Intra-class correlation coefficients (ICCs) were used to evaluate the intra- and inter-observer agreement in terms of feature extraction. We interpreted an ICC of 0.81–1.00 as almost perfect agreement, 0.61–0.80 as substantial agreement, 0.41–0.60 as moderate agreement, 0.21–0.40 as fair agreement, and 0–0.20 as poor or no agreement. An ICC above 0.6 was considered a mark of satisfactory inter- and intra-observer reproducibility.

### Radiomics feature extraction

**Fig. 1:**
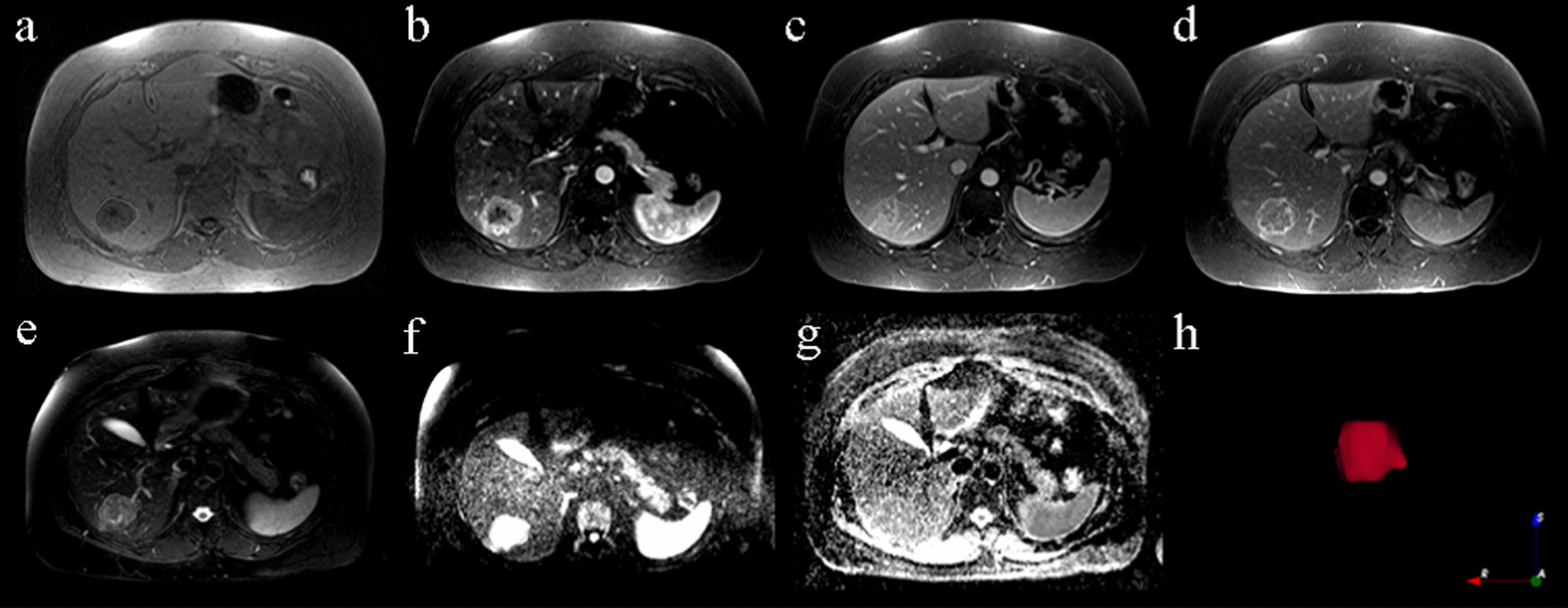
An example of the tumor VOI for different MRI sequences. The VOI were delineated on T2WI, then DWI and ADC sequences shared the same VOI after being registered to T2WI.In addition, T1WI, Portal venous phase and Delay phase sequences shared the same VOI after being registered to Arterial phase. **a** TIWI, **b** arterial phase, **c** portal venous phase, **d** delay phase, **e** T2WI, **f** DWI (b = 800), **g** ADC, **h** a three-dimensional rendering of the tumor region

Potential impact of scanning parameters, protocols, scanners, and vendors was minimized by normalizing all MRI sequences using Z-scores before feature extraction. Volume of interest (VOI) of tumor was manually delineated slice-by-slice on T2WI and T1WI arterial phase by three experienced radiologists using MRIcrogL (v.1.2.20210317, https://www.nitrc.org/projects/mricrogl/). The first radiologist delineated images of 21 patients in 2019, whereas the second and third radiologists delineated images of 20 patients each in 2020 and 2021, respectively. Any problematic delineations were reviewed and modified by an experienced senior medical radiologist. In-house software based on 3Dslicer (v. 4.10.2 r28257; https://www.slicer.org/) was used to align the ADC and DWI (b = 800) images to the T2WI using rigid registration and trilinear interpolation, Obtaining the same resolution, space, and origin. Afterward, the T1WI arterial phase and T1WI portal venous phase were aligned to the T1WI venous phase. As illustrated in Fig. 1, each sequence shows a tumor volume of interest. By defining VOI in advance, any MRI sequence could use the outlined segment. We quantized the full range of intensity in VOI into 32 different gray levels in order to extract features.

Pyradiomics (v. 2.1.0), an open-source Python package for detecting radiomic features in medical images, was used to extract VOIs from seven types of MRI sequences. A total of seven categories of radiomics features were extracted: 19 first-order statistics features; 16 shape-based features; 24 Gy level cooccurrence matrix (GLCM) features; 16 Gy level run length matrix (GLRLM) features; 16 Gy level size zone matrix (GLSZM) features; 5 neighboring gray tone difference matrix (NGTDM) features; and 14 Gy level dependence matrix (GLDM) features. All radiomics features except shape were computed using 7 different filters, including gradient, wavelet, logarithm, square, square root, exponential, and local binary pattern in 3D (LBP3D). There were 1782 radiomic features in total extracted from each VOI.

### Feature selection and classifier modeling

**Fig. 2:**
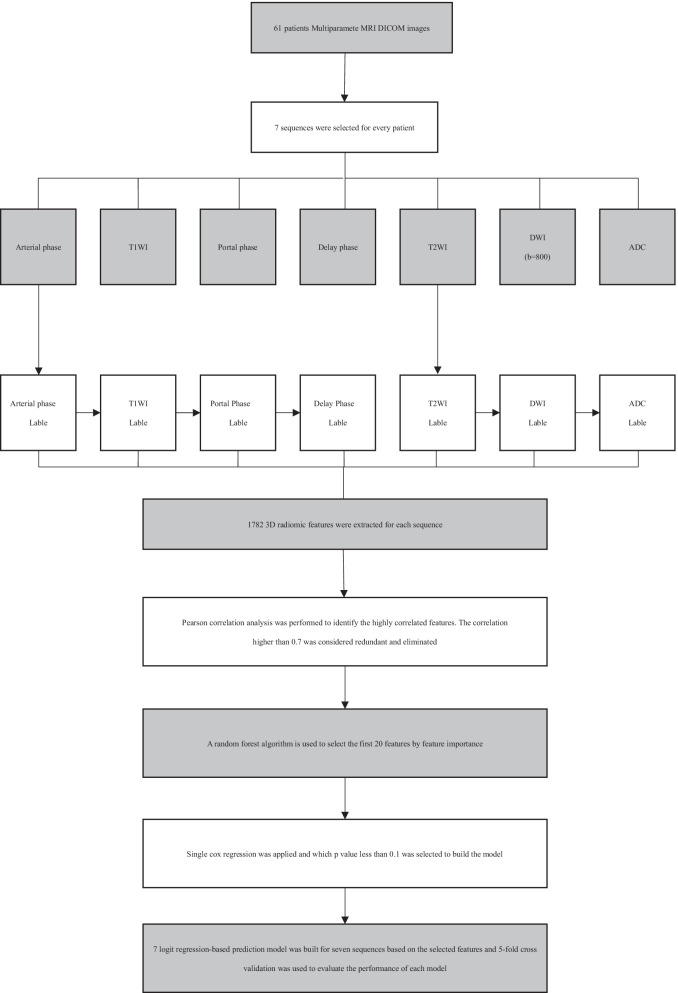
Framework for radiomic analysis

The feature selection process was used to ensure model accuracy and to avoid overfitting. This also ensured model accuracy and avoided overfitting. Three steps were used to feature dimension reduction for seven sequences. Firstly, For the purpose of identifying highly correlated features, a Pearson correlation analysis was conducted. A correlation > 0.7 was considered redundant and was eliminated. Secondly, The top 20 features were selected using the random forest algorithm based on feature importance. Finally, single cox regression was applied, in which p < 0.1 was selected to build a model. On the basis of selected features, 7 logit regression models were constructed for 7 sequences, and performance was assessed using fivefold cross validation (Fig. 2). In addition, a clinical prediction model based on clinical characteristics was built. MRI radiomics signatures were combined with clinical characteristics to evaluate the impact of clinical indicators.

### Statistical analysis

A set of independent tests was conducted on the Radiomics signatures. AUCs and accuracy were determined using receiver operating characteristic curves (ROC curves). Based on a positive likelihood ratio, a cutoff value was determined to determine the sensitivity and specificity of the test. Clinical indicators were used in univariate and multivariate analyses to identify independent prognostic factors associated with PFS based on the Cox regression model.

MRI radiomics signature and a subset of clinical characteristics were combined based on Akaike information criterion (AIC). For purposes of deleting or adding variables, the smallest information statistics were selected. R software (4.0.3, Vienna, Austria) was used to conduct feature selection, classifier modeling, and statistical analysis, with p < 0.05 defining statistical significance.

## Results

### Characteristics of study cohorts

During follow-up, 39 patients (64.0%) developed progressive disease with a median follow-up period of 12 months (range, 3–25 months) in the present study. Detailed baseline characteristics of all patients are presented in Tables 1 and 2. Among the 61 patients, CR, PR, SD were achieved in 14 (23.0%), 35 (57.4%) and 7 (11.5%) patients, respectively. Overall ORR was 80.3%, whereas overall DCR was 91.8% (Table 3).

**Table 1:** Characteristics of the study patients

Clinical characteristic	Patient (n = 61)
*Age*	
≥ 65	25
< 65	36
*Gender*	
Male	55
Female	6
*Weight*	
≥ 60 kg	23
< 60 kg	38
*BCLC*	
A	3
B	19
C	39
*Child–Pugh*	
A	55
B	6
*Tumor numbers*	
1	12
≥ 2	39
Tumor size (cm)	
*HBV*	
Positivity	57
Negative	4
*AFP (ng/ml)*	
≥ 400	32
< 400	29
*GGT (U/L)*	
≥ 60	45
< 60	16
*ALP (U/L)*	
≥ 125	26
< 125	35
*AST (U/L)*	
≥ 40	46
< 40	15
*ALT (U/L)*	
≥ 50	34
< 50	27
ALB (g/L)	39.1 ± 5.2
TBIL (µmol/L)	18.9 ± 15.0
PT (s)	12.4 ± 1.2
NLR	3.0 ± 1.9
LMR	4.0 ± 2.1
PLR	154.6 ± 76.7
PNI	47.8 ± 7.3
SII	671.1 ± 565.6

AFP, alpha-fetoprotein; AST, aspartate aminotransferase; ALP, alkaline phosphatase; ALT, Alanine aminotransferase. ALB, Albumin; BCLC, Barcelona Clinic Liver Cancer; HBV, hepatitis B virus; Child–Pugh; GGT, γ-glutamyl transpeptidase;LMR, lymphocyte to monocyte ratio; NLR, neutrophil to lymphocyte ratio; PLR, platelet to lymphocyte ratio; PNI, prognostic nutritional index; PT, Prothrombin time; SII, systemic immune-inflammation index; TBIL, total bilirubin

**Table 2:** Early treatment response evaluation using mRECIST 4–12 weeks after initiation of treatment in all patients and by BCLC stage

Category	Response evaluation using mRECIST				ORR (%)	DCR (%)
	CR	PR	SD	PD		
ALL case (n = 61)	14	35	7	5	80.3	91.8
BCLC stage A/B (n = 22)	2	14	4	2	72.7	90.9
BCLC stage C (n = 39)	12	21	3	3	84.6	92.3

Overall objective response rate (ORR) was defined as the percentage of patients with a best overall response of complete response (CR) or partial response (PR). Disease control rate (DCR) was defined as the percentage of patients with a best overall response of CR, PR, or stable disease (SD)

CR = complete response; SD = stable disease; PR = partial response; PD = progressive disease

**Table 3:** MRI scanning parameter

Scanners	GE medical systems (3.0 T)	Siemens	UIH
	N = 27	N = 20	N = 14
T_1_ T_E_ (ms)	1.4–2.4	2.0	1.4
T_1_ T_R_ (ms)	3.9–4.9	4.4	3.7
T_1_ slice thickness (mm)	5.0	5.0	5.0
T_2_ T_E_ (ms)	90	80	70
T_2_ T_R_ (ms)	2200–3000	2000	2400
T_2_ slice thickness (mm)	7.0	6.0	7.0
FOV (mm)	384 × 286	380 × 269	375 × 306

### Predictive factors related to PFS

Multivariate analysis using a stepwise Cox hazard model revealed that tumor number (hazard ratio, HR = 4.64, [95% CI 1.03–20.88], P = 0.045), and intensity enhancement on arterial phase (HR = 0.24, [95% CI 0.09–0.64], P = 0.004) were independent risk factors for disease progression (Table 4).

**Table 4:** Univariate and multivariate analysis of clinical factors associated with progression events

Characteristic	Univariate			Multivariate		
	HR	95%CI	*P* value	HR	95%CI	*P* value
Age	1.00	(0.97–1.0)	0.6			
Sex	4.2e−10	(0-Inf)	1			
Tumor size	1.00	(0.93–1.1)	0.59			
Tumor number	3.70	(1.1–12.0)	0.035*	4.64	1.03–20.88	0.045
HBV	0.33	(0.1–0.99)	0.048*			
AFP	0.94	(0.42–2.1)	0.88			
GGT	1.10	(0.42–2.7)	0.89			
ALP	0.83	(0.38–1.8)	0.65			
AST	0.58	(0.24–1.4)	0.23			
Child–Pugh	1.00	(0.23–4.3)	0.99			
BCLC	1.40	(0.63–3.0)	0.42			
PLR	1.50	(0.66–3.4)	0.33			
LMR	1.80	(0.76–4.2)	0.18			
PNI	1.30	(0.55–3.0)	0.57			
SII	1.70	(0.75–3.8)	0.21			
NLR	0.62	(0.21–1.8)	0.39			
Location	1.70	(0.80–3.8)	0.16			
Tumor margin	1.00	(0.44–2.4)	0.93			
Types of gross growth	1.80	(1.1–3.0)	0.038*	1.15	0.6–2.17	0.676
ADC intensity display	1.00	(0.11–2.4)	0.93			
Signal homogeneity on T2WI	0.82	(0.36–1.8)	0.63			
Intensity enhancement on arterial phase	0.35	(0.15–0.8)	0.013*	0.24	0.09–0.64	0.004
Expansion of arterial phase range	0.35	(0.15–0.8)	0.81			
Type of enhancement	0.56	(0.24–1.3)	0.17			
Arterial peritumoral enhancement	0.86	(0.35–2.1)	0.73			
Enhancing tumor capsule	0.77	(0.35–1.7)	0.53			
Hepatic capsule bulge	0.54	(0.24–1.2)	0.14			
Scarring centrally	0.34	(0.08–1.5)	0.15			
Hemorrhage	0.53	(0.07–4.2)	0.55			
Necrosis	2.20	(0.91–5.5)	0.079			
Fat	0.22	(0.03–1.8)	0.15			
Massive vein tumor thrombosis	0.85	(0.44–1.6)	0.62			
Metastasis in distant areas	1.76	(0.83–3.7)	0.14			

HBV was not selected. Because only 4 patients did not have hepatitis B. Based on clinical considerations, it was not subjected to multivariate analysis

*P < 0.05

### Predictive performance of nine models

A clinical model contains conventional preoperative factors and features, whereas the combined model incorporates the radiomics signature (7 series) into clinical characteristics. The Table 5 shows the ROCs and AUCs of nine radiomics signatures. With a sensitivity of 0.27 and specificity of 0.95, the AUC of the clinical model was 0.71.

**Table 5:** Predictive performance of the radiomics signature and clinical model

Varibales and models	Sensitity	Specificity	Accuracy
Clinical model	0.27	0.95	0.71
T1WI	0.45	0.8	0.68
T1WI arterial phase	0.01	0.95	0.61
T1WI portal venous phase	0.64	0.85	0.77
T1WI delay phase	0.27	1	0.74
T2WI	0.55	0.9	0.77
ADC	0.54	0.85	0.76
DWI(b = 800)	0.72	0.45	0.55
Combined models	0.99	0.95	0.71

As a result of integrating radiomics signature with conventional variables, there was a significant improvement in precision of clinical models as assessed by AUC of 0.71, sensitivity of 0.99, and specificity of 0.95.

**Fig. 3:**
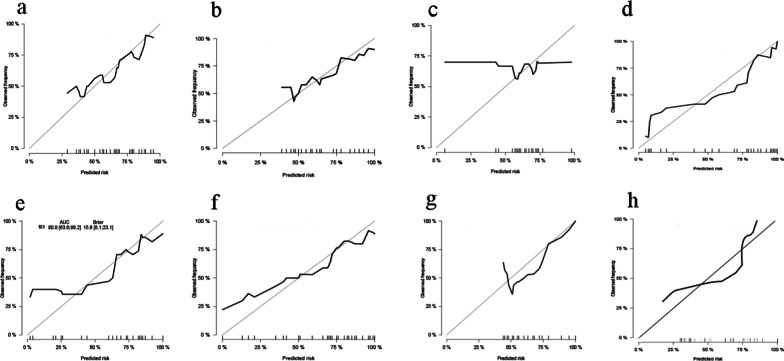
Calibration curve of each model. **a** T1WI model; **b** T1WI arterial phase model; **c** T1WI portal venous phase model; **d** T1WI delay phase model; **e** T2WI model; **f** ADC model; **g** DWI model; **h** combined models

Figure 3 shows the Calibration curve of each model.

## Discussion

The effectiveness of chemotherapy in HCC is restricted by chemo-resistance and systemic side effects [17]. The current study showed that HCC MRI image features such as tumor numbers and intensity enhancement on arterial phase independently affected PFS. The combined model used in the current study demonstrated improved performance compared with clinical models.

Previous studies have recently reported some biomarkers for predicting efficacy of Lenvatinib therapy, although most of them were predictive of survival [18–20]. Median survival was not reported as the current study focused on time to PFS. In addition, few studies on biological indicators of short-term efficacy of combined Lenvatinib and TACE therapy have been undertaken. Furthermore, exploratory studies focusing on the role of MRI in predicting combined treatment of PFS have not been undertaken.

The current study established that patients with single liver cancer had longer progression-free survival time than patients with multiple liver cancers (HR = 4.64, [95% CI 1.03–20.88]), which is consistent with findings of similar previous studies [3]. This may be explained by the fact that biological characteristics of multi-nodular liver cancer are more aggressive than those of single liver cancer. In addition, multi-nodular liver cancer is easier to recur and metastasize compared with single liver cancer.

Notably, intensity enhancement on the arterial phase was associated with poor response to combined therapy. If arterial phase intensity enhancement was less than portal vein intensity enhancement, the progression-free survival rate would decrease by 76%. This is because of poor tumors perfusion, which was associated with high HCC marker expression, such as vascular endothelial growth factor A (VEGFA) and Fibroblast Growth Factor Receptor 4 Level (FGRF4) [21, 22]. This relationship may be explained by tumor progression, which results in decreased arterial flow, because higher interstitial pressure leads to closing of arterial capillaries. Negative correlations between VEGFA expression and perfusion have been suggested by hypoxia induced expression of VEGFA in a hypoxic environment. As a result of its interaction with mainly VEGFA, lenvatinib has been identified as antiangiogenic. Because of this, it may be more ineffective in tumors with inadequate blood supply. Hypovascular HCC has a poor arterial blood supply, so it is difficult to control with conventional TACE, the TACE efficacy was low[23–25]. Kawamura [26] evaluated 51 patients with unresectable liver cancer who received continuous Lenvatinib treatment. Dynamic CT analysis showed that tumor response rates for HCC nodules with a heterogeneous enhancement pattern are significantly high. Despite this, heterogeneity of dynamic CT enhancement pattern did not demonstrate differences in PFS.

Use of radiomics signatures in the current study established that clinical models can be improved by stratifying patients by their image features, which could improve individual treatment. According to the results of the current study, patients at high risk of progression should consider alternative treatment strategies and preoperative adjunctive therapies, which are consistent with the results of a previous study [27] that focused on predicting short-term recurrence of liver cancer using CT-enhanced sequences after surgical resection. In a retrospective analysis of 67 patients with HCC, Shusuke [28] et al. found that contrast enhanced CT could predict the outcome of Lenvatinib treatment, However, the study lacks external validation. With the popularity of MRI in clinical practice, the use of MRI-based radiomic features can help clinicians formulate more accurate HCC prognosis, according to recent studies [29–32]. Patients with poor PFS screened by this model are treated with Regorafenib [33] or PD1 [34]. Of the 61 patients, only 6 have died so far. This implies greater benefit from their use, indicating greater reliability and better prediction performance. It is important to validate the preliminary nomogram with a larger sample size in order to make sure it is useful and to minimize overfitting.

### Study limitations

Retrospective studies are prone to selection bias. In addition, the current study had a relatively small patient population and a low percentage of patients who had progressive disease. Although Lenvatinib combined with TACE in the treatment of advanced liver cancer is also used in other hospitals, there are few studies based on MRI radiomics [28], and we also lack externally validated data. Therefore, this article did not conduct external validation. We are working with other hospitals to conduct external verification and are currently undergoing an ethical review. A correct standardization of the procedures it is also possible to reduce the number of rays absorbed for both patients and radiologists and reduce possible stochastic effects [35].We did not consider accurately the amount of absorbed radiant dose, because each patient's blood vessel condition during the operation is different, the operation time will be different, and the radiation dose will also be different. We will record the radiation dose for each operation in future studies. Furthermore, only radiomic features of the HCC tumors were examined in the current study. Prediction of progression risk can be undertaken by imaging neighboring tissue around nodules. However, biological mechanisms by which these image features become apparent in radiomics studies are still obscure. Therefore, further studies on mechanistic insights into these radiomics features are needed in order to investigate possible correlations between radiomics and tumor genomics or proteomics.

## Conclusions

The current study demonstrated that Lenvatinib plus TACE is feasible for advanced unresectable HCC with a positive therapeutic response, although there is a significant early progression risk. A high number of tumors on MRI before treatment and low arterial phase intensity enhancement after treatment were independent markers of disease progression. In addition, combined MRI-based radiomics features achieved superior prognostic model.

## Data Availability

The corresponding author can provide data supporting the conclusions of this study upon reasonable request. The authenticity of this article has been validated by uploading the key raw data onto the Research Data Deposit public platform (www.researchdata.org.cn), with the approval RDD number as RDDA2022667871.
